# Ban fluorinated organic substances to spark green alternatives

**DOI:** 10.1016/j.eehl.2022.07.001

**Published:** 2022-08-02

**Authors:** Christian Sonne, Changlei Xia, Su Shiung Lam

**Affiliations:** a Aarhus University, Faculty of Technological Sciences, Department of Ecoscience, DK-4000 Roskilde, Denmark; b Sustainability Cluster, School of Engineering, University of Petroleum & Energy Studies, Dehradun, Uttarakhand 248007, India; c Jiangsu Co-Innovation Center of Efficient Processing and Utilization of Forest Resources, International Innovation Center for Forest Chemicals and Materials, College of Materials Science and Engineering, Nanjing Forestry University, Nanjing 210037, China; d Universiti Malaysia Terengganu, Higher Institution Centre of Excellence (HICoE), Institute of Tropical Aquaculture and Fisheries, 21030 Kuala Nerus, Malaysia; e Academy of Sciences Malaysia, Young Scientists Network, Top Research Scientists Malaysia (TRSM), 50480 Kuala Lumpur, Malaysia

**Figure:**
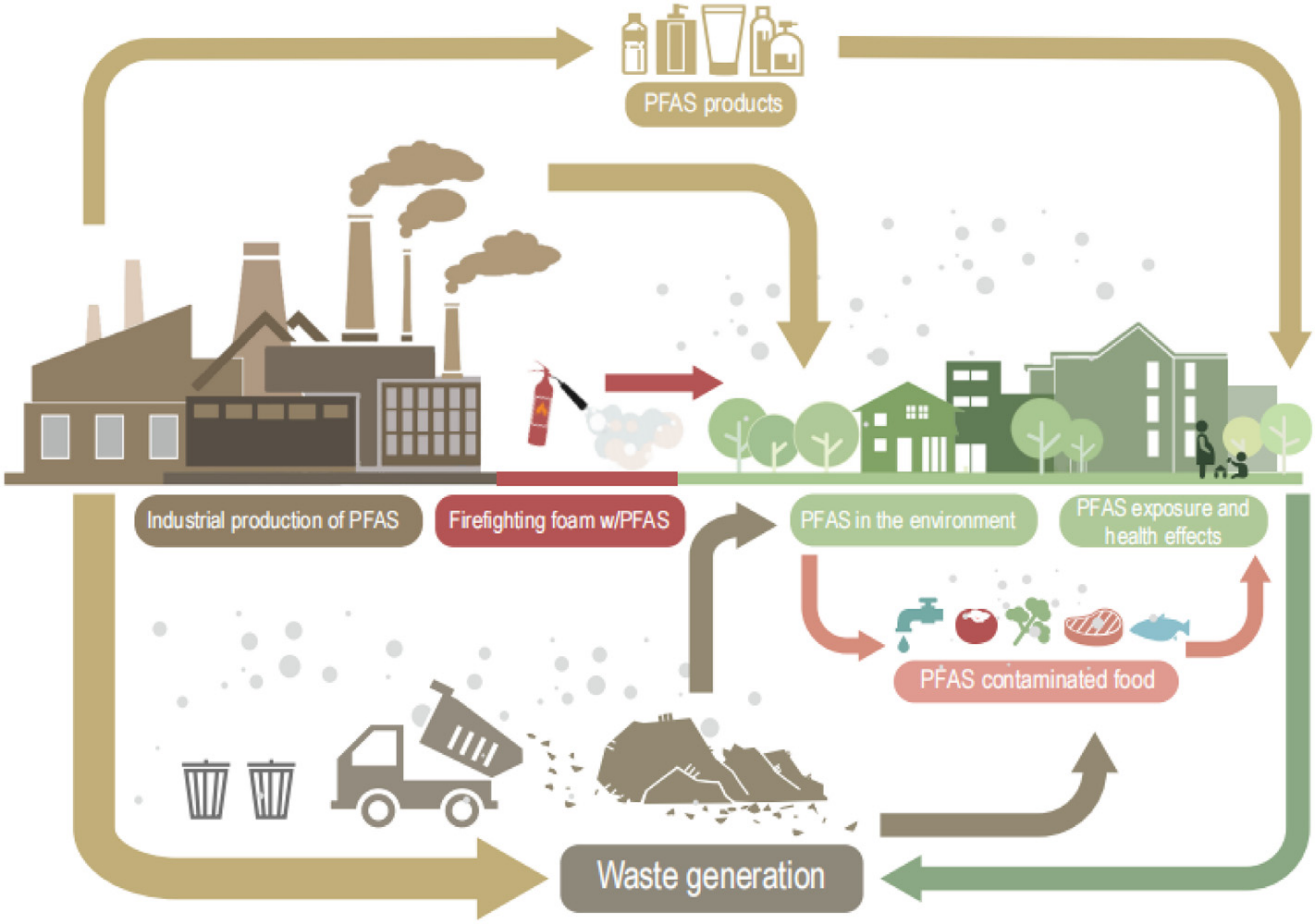

**Per- and polyfluoroalkyl substances (PFAS)** do not readily biodegrade and are therefore routinely found in the environment as ubiquitous contaminants in wildlife and humans worldwide [1,2]. These “forever chemicals” are among the major chemical threats to wildlife and human health nowadays and are used in consumer products, including waterproof rain wear, pizza boxes, Teflon, and firefighting foam [3,4]. Due to their resistance to metabolism and excretion biomagnifying in food webs to high concentrations [5,6], PFAS can bind to proteins and affect the immune system that increase the risk of clinical and infectious diseases and autoimmunity [[7], [8], [9]].

In their review, M. G. Evich et al. [10] showed that over the past few years, thousands of toxic PFAS have been used globally and entered the environment through the atmosphere and aquatic pathways, hence calling for remediation of drinking water especially. Regrettably, there is more to the problem than remediation, as PFAS replacement chemicals also persist in the environment. Such emerging compounds, including the non-regulated F-53B, Gen-X, and ADONA that replace some of the widely used PFAS, undergo long-range oceanic or atmospheric transport to the Arctic where they biomagnify and threaten wildlife conservation [6]. Similar transboundary ecosystem contamination has been reported in the US as well [11].

Thousands of new chemicals are developed each year, and around 400 million tons of chemicals in total are produced yearly on a global scale, with a large part ending up in the environment and human body [12]. The yearly historical PFAS production worldwide of these PFAS is estimated to be around 100,000 tons, reflecting that the current replacement strategy by the Stockholm Convention does not meet the necessities of mitigation of toxic PFAS in the environment [13,14]. This urges a complete ban on fluorinated compounds in pizza card boxes, aluminum foil, dental floss, cosmetics, textiles, Teflon, and firefighting foam to mention a few [15,16]. Unfortunately, too few non-fluorinated alternatives exist in the market, calling for urgent research on such materials’ development. Using nanocellulose for food packaging is one example of adopting non-toxic raw materials, and so is halogen-free firefighting foam derived from complex carbohydrates [17,18]. One obvious way could be to ban the use of all fluorinated compounds in food packaging and daily consumer products, including make-up and other cosmetics, to reduce human exposure risks and environmental pollution. A second step should follow the needed spark in innovative research on sustainable (greener) alternatives to facilitate a complete phase-out over the coming decade. Such a priority is needed since we need time to adjust the production of chemicals with widespread industrial applications and implement the phase-out of old substances and the phase-in of new ones. Considering the excellent physical, chemical properties of PFAS, a complete global ban needs to be taken stepwise to ensure a realistic plan of the phase-out of these toxic and persistent substances, which is urgently warranted.

Without swift action, these long-lasting chemicals will continue to threaten the environment and human health because of their carcinogenic, endocrine-disruptive, and immunotoxic properties [19,20]. The EU, US EPA, and the Ministry of Ecology and Environment of China need to do more by learning from Denmark and the state of Maine, and initiate a complete global ban on PFAS through the Stockholm Convention, while investing in alternative sustainable biological-inspired less-toxic compounds. Otherwise, it is not possible to meet the SDG 3.9 that targets environmental pollution abatement and planetary health [21,22].

## Declaration of competing interests

The authors have declared no conflicts of interest.
